# Complete genome sequence of Thermus brockianus GE-1 reveals key enzymes of xylan/xylose metabolism

**DOI:** 10.1186/s40793-017-0225-7

**Published:** 2017-02-03

**Authors:** Christian Schäfers, Saskia Blank, Sigrid Wiebusch, Skander Elleuche, Garabed Antranikian

**Affiliations:** Institute of Technical Microbiology, Hamburg University of Technology (TUHH), Kasernenstraße 12, 21073 Hamburg, Germany

**Keywords:** Whole genome sequence, *de novo* assembly, *Thermus*, *Thermus brockianus*, Xylan degradation, Xylose metabolism, Thermophiles, Single molecule real-time sequencing

## Abstract

*Thermus brockianus* strain GE-1 is a thermophilic, Gram-negative, rod-shaped and non-motile bacterium that was isolated from the Geysir geothermal area, Iceland. Like other thermophiles, *Thermus* species are often used as model organisms to understand the mechanism of action of extremozymes, especially focusing on their heat-activity and thermostability. Genome-specific features of *T. brockianus* GE-1 and their properties further help to explain processes of the adaption of extremophiles at elevated temperatures. Here we analyze the first whole genome sequence of *T. brockianus* strain GE-1. Insights of the genome sequence and the methodologies that were applied during *de novo* assembly and annotation are given in detail. The finished genome shows a phred quality value of QV50. The complete genome size is 2.38 Mb, comprising the chromosome (2,035,182 bp), the megaplasmid pTB1 (342,792 bp) and the smaller plasmid pTB2 (10,299 bp). Gene prediction revealed 2,511 genes in total, including 2,458 protein-encoding genes, 53 RNA and 66 pseudo genes. A unique genomic region on megaplasmid pTB1 was identified encoding key enzymes for xylan depolymerization and xylose metabolism. This is in agreement with the growth experiments in which xylan is utilized as sole source of carbon. Accordingly, we identified sequences encoding the xylanase Xyn10, an endoglucanase, the membrane ABC sugar transporter XylH, the xylose-binding protein XylF, the xylose isomerase XylA catalyzing the first step of xylose metabolism and the xylulokinase XylB, responsible for the second step of xylose metabolism. Our data indicate that an ancestor of *T. brockianus* obtained the ability to use xylose as alternative carbon source by horizontal gene transfer.

## Introduction

Members of the genus *Thermus* are Gram-negative, rod-shaped, non-sporulating, thermophilic aerobic bacteria. They have been discovered from various environments with elevated temperatures, including hot springs, deep-sea hot vents, volcanic eruptions and solfatara fields [1–4]. *Thermus aquaticus* was first isolated in 1969 in hot springs in Yellowstone National Park, USA [5]. *Thermus* species and their produced enzymes, so called extremozymes, have attracted the attention of scientists from academia and industry due to their unique properties and metabolic pathways. Robust biocatalysts are attractive to various applications that often prevail in industrial processes [6–8]. The most prominent example of an industrial-relevant extremozyme is the DNA polymerase from *T. aquaticus* that is applied in polymerase chain reaction. Further industrial applications using enzymes from *Thermus* species include laundry detergents, DNA clean up prior to PCR or C-terminal sequencing [9–12]. Recently two glycoside hydrolases from *T. antranikianii* and *T. brockianus* were described and extended this group of industrial-relevant enzymes [13].

*T. brockianus* strain GE-1 was chosen for whole genome sequencing due to its ability to use xylan as sole carbon source and degrade xylan-rich substrates (Blank and Antranikian, unpublished results) [14]. To our knowledge the hydrolysis of xylan has not been described for any other *T. brockianus* strain so far, including type strain YS038^T^ [15]. With the description of the corresponding thermostable xylanase, Xyn10, we already identified and characterized one of the key enzymes in a putative xylan degradation pathway of *T. brockianus* GE-1 [14]. The identification and characterization of other polymer degrading enzymes from *Thermus* species is of great interest since there are only few reports regarding this aspect [16, 17]. Especially in the view of finding new solutions for global challenges like degradation of xenobiotic compounds or providing novel renewable energy sources, the xylanolytic behavior of *T. brockianus* GE-1 justifies further examination. These findings will also contribute to the development of biotechnological processes based on lignocellulose as carbon source (biorefinery). In this paper we present the first whole genome sequence of a *T. brockianus* strain with finished grade status, showing a phred quality value of QV50.

## Organism information

### Classification and features

**Fig. 1:**
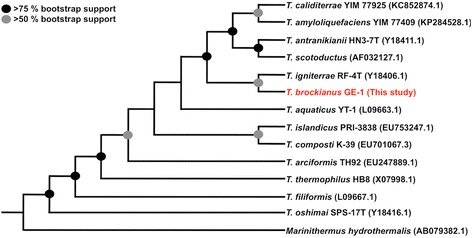
Unrooted phylogenetic tree based on 16S rRNA encoding sequences from 14 species of the genus *Thermus*. The phylogenetic tree was generated using the program package PHYLIP (version 3.695) [58] and TreeView X [59], based on a multiple sequence alignment (1,345 nts) that was generated with clustalX [60]. The number of nucleotide replacements at each position in the sequence was estimated with the DNADIST program and trees were constructed using NEIGHBOR. Bootstrap analysis was done using 1,000 iterations. CONSENSE was used to produce a majority rule consensus tree. The position of the isolate *Thermus brockianus* strain GE-1 is indicated in red. The 16S rRNA encoding sequence from *Marinithermus hydrothermalis* was used as outgroup. Accession numbers of all sequences are indicated in the figure. For the following species sequenced genomes are available at NCBI (number of available genome sequences are given in square brackets): *T. caliditerrae* [1], *T. amyloliquefaciens* [1], *T. antranikianii* [1], *T. scotoductus* [4], *T. igniterrae* [1], *T. brockianus* [1, this study], *T. aquaticus* [3], *T. islandicus* [1], *T. thermophilus* [5], *T. filiformis* [1], *T. oshimai* [2] and *M. hydrothermalis* [1]

*T. brockianus* type strain YS038^T^ has been described as a member of the family *Thermaceae* within the phylum *Deinococcus-Thermus*. The isolate GE-1 could be clearly assigned to the species *T. brockianus* based on sequence information and 16S rRNA pairwise alignment, indicating 100% identity in 1,476 nucleotides overlap. The phylogenetic classification of *T. brockianus* GE-1 within the *Deinococcus-Thermus* group is displayed in the phylogenetic tree shown in Fig. 1. Further alignments with closely related species of the genus *Thermus* were performed using the webserver LALIGN [18] and revealed the following results: *T. igniterrae* strain RF-4 T (96.8% identity in 1,477 nts overlap), *T. aquaticus* strain YT-1 (96.1% identity in 1,474 nts overlap), *T. composti* strain K-39 (96.1% identity in 1,445 nts overlap), *T. islandicus* strain PRI-3838 (95.9% identity in 1,445 nts overlap), *T. arciformis* strain TH92 (95.7% identity in 1,484 nts overlap), *T. caliditerrae* strain YIM 77925 (95.0% in 1,514 nts overlap), *T. amyloliquefaciens* strain YIM 77409 (94.9% identity in 1,513 nts overlap), *T. scodoductus* (94.9% identity in 1,476 nts overlap), *T. thermophilus* strain HB8 (94.9% identity in 1,518 nts overlap), *T. antranikianii* strain HN3-7 T (94.7% identity in 1,482 nts overlap), *T. filiformis* (94.0% in 1,475 nts overlap) and *T. oshimai* SPS-17 T (91.4% identity in 1,477 nts overlap).

**Fig. 2:**
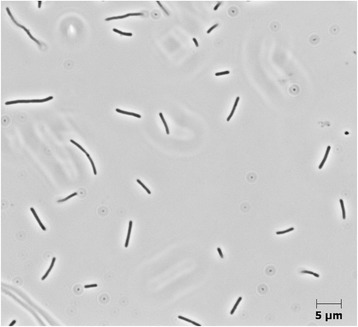
Photomicrograph of *T. brockianus* GE-1

*T. brockianus* strain GE-1 is a Gram-negative, rod-shaped, non-pathogenic and non-sporulating bacterium (Fig. 2). This strain is aerobic, yellow pigmented and non-motile (Table 1). Due to their thermophilic characters, *Thermus* species are capable of thriving at elevated temperatures in a range between 45 °C and 83 °C and most species show an optimal growth at 80 °C [19]. While most of the species within the *Thermus* genus were reported to degrade a diverse set of sugars, including D-glucose, D-fructose, D-galactose, D-ribose, lactose and sucrose, only a few strains such as *T. brockianus* YS038^T^ or *T. thermophilus* HB8 were described to utilize D-xylose [15]. However growth on xylan was only reported for the strain *T. brockianus* GE-1 and a corresponding xylanase-encoding gene has been identified within its genome (Blank and Antranikian, unpublished results) [14]. *T. brockianus* strain GE-1 was isolated from the Geysir geothermal area, Iceland. It optimally grows at a temperature of 70 °C and 160 rpm. The minimum information about the genome sequence (MIGS) [20] of that strain is shown in Table 1.

**Table 1:** Classification and general features of *T. brockianus* GE-1 according to MIGS [20]

MIGS ID	Property	Term	Evidence code^a^
	Classification	Domain *Bacteria*	TAS [48]
		Phylum *Deinococcus-Thermus*	TAS [49, 50]
		Class *Deinococci*	TAS [51, 52]
		Order *Thermales*	TAS [51, 53]
		Family *Thermaceae*	TAS [51, 54]
		Genus *Thermus*	TAS [5, 49, 55]
		Species *Thermus brockianus*	TAS [56]
		Strain GE-1	IDA
	Gram stain	Negative	IDA
	Cell shape	Rod	IDA
	Motility	Non-motile	NAS
	Sporulation	Non-sporulating	NAS
	Temperature range	45-83 °C	TAS [19]
	Optimum temperature	70 °C	TAS [19]
	pH range; Optimum	pH 7.0 – pH 8.0	NAS
	Carbon source	Diverse set of sugars	IDA
MIGS-6	Habitat	Terrestrial hot springs	IDA
MIGS-6.3	Salinity	Not reported	
MIGS-22	Oxygen requirement	Aerobic	NAS
MIGS-15	Biotic relationship	Free-living	NAS
MIGS-14	Pathogenicity	Non-pathogen	NAS
MIGS-4	Geographic location	Geysir geothermal area, Iceland	IDA
MIGS-5	Sample collection	1992	IDA
MIGS-4.1	Latitude	Not reported	-
MIGS-4.2	Longitude	Not reported	-
MIGS-4.4	Altitude	Not reported	-

^a^Evidence codes - IDA: Inferred from Direct Assay; TAS: Traceable Author Statement (i.e., a direct report exists in the literature); NAS: Non-traceable Author Statement (i.e., not directly observed for the living, isolated sample, but based on a generally accepted property for the species, or anecdotal evidence). These evidence codes are from the Gene Ontology project [57]

## Genome sequencing information

### Genome project history

We conceived the whole *de novo* genome sequencing of *T. brockianus* GE-1 because of its ability to degrade xylan-rich biomass that has not been described for any other *T. brockianus* species so far and thus it has great potential for application in future biorefineries. Comparison of its genome sequence to that of other sequenced *Thermus* species will also help to understand general molecular features of xylan degradation in thermophiles. Sequencing was done at GATC Biotech AG (Konstanz, Germany). The *de novo* assembly, annotation and finishing of the whole genome of *T. brockianus* GE-1 was performed at the Institute of Technical Microbiology at Hamburg University of Technology (TUHH). The finished genome sequence, including three circular replicons, has been submitted to National Center of Biotechnology Information (NCBI) in June 2016. A summary of the project information is shown in Table 2.

**Table 2:** Project information

MIGS ID	Property	Term
MIGS 31	Finishing quality	Finished genome
MIGS-28	Libraries used	PacBio RS library
MIGS 29	Sequencing platforms	PacBio RS II
MIGS 31.2	Fold coverage	156.56x PacBio
MIGS 30	Assemblers	HGAP2 version 2.3.0
MIGS 32	Gene calling method	Prodigal v2.6
	Locus Tag	A0O31
	Genbank ID	CP016312, CP016313, CP016314
	Genbank Date of Release	November 17, 2016
	GOLD ID	Gp0134387
	BIOPROJECT	PRJNA314486
MIGS 13	Source Material Identifier	GE_001
	Project relevance	Biotechnological

### Growth conditions and genomic DNA preparation

*T. brockianus* strain GE-1 was obtained from the strain culture collection of the Institute of Technical Microbiology at Hamburg University of Technology (TUHH). Deposition of the strain in the German National Culture Collection (DSMZ) is in progress. The strain was grown aerobically in DSMZ medium 878 (*Thermus* 162 medium) at 70 °C for at least 48 h and agitation speed of 160 rpm [19]. The genomic DNA of *T. brockianus* GE-1 was isolated using the PowerSoil DNA Isolation Kit (Mobio, USA). All steps were performed according to the manufacture’s instructions. Quality control of the isolated DNA was checked at GATC Biotech AG (Konstanz, Germany) prior to sequencing. A DNA concentration of 83.1 ng/μl and a 260/280 ratio of 1.87 were determined.

### Genome sequencing and assembly

Third generation sequencing technology from Pacific Biosciences was chosen for whole *de novo* genome sequencing of *T. brockianus* strain GE-1 because its continuous long reads of up to 10 kb covering the longest known bacterial and archaeal repetitive regions and thus facilitate the generation of complete bacterial genome assemblies [21, 22]. Library construction, quantification and sequencing were performed at GATC Biotech AG (Konstanz, Germany). A Pacbio RS library (8–12 kb) was constructed and one SMRT cell was used for sequencing. 86,479 subreads were obtained after filtering and a total of 447.6 Mb with a N50 contig length of 2,058,948 bp were used for assembly. Pacific Biosciences sequencing data were assembled using an implemented version of PacBio SMRT Analysis, version 2.3.0 and the HGAP2 protocol (Pacific Biosciences, USA) [22]. Minimum seed read length was automatically determined by the protocol with a length cut-off of 10,819 bp. The sum of contig lengths was 2,431,825 bp. The final *de novo* assembly obtained three circular contigs, providing the complete genome sequence of *T. brockianus* strain GE-1 and genome coverage of 156.56. Each contig represented one replicon, including the chromosome (2,035,182 bp), the megaplasmid pTB1 (342,792 bp) and plasmid pTB2 (10,299 bp). Circularization of each replicon was checked and performed by using circlator [23]. Quality value of > QV50 (1 error probability in 100,000 base calls) for each replicon was reached after several polishing steps using the quiver algorithm, included in PacBio SMRT Analysis, version 2.3.0 [22].

### Genome annotation

Preliminary genome annotation was performed using the Prokka annotation pipeline v1.12 [24], followed by manual curation. Genes were identified by both Prodigal v2.6.3 [25] and Glimmer v3.0.2 [26]. Predicted coding sequences were translated and used to search the NCBI non-redundant database [27], UniProt [28] and Pfam [29] databases. The cmmscan and cmmsearch tools of the Hmmer3 package were used for protein similarity searches against HMM databases [30]. For COG classification RPS-BLAST was used to search against the COG database [31]. For rRNA detection we used RNAmmer v1.2 [32], while the tRNA prediction was performed by tRNAscan-SE v1.3.1 [33]. Non-coding RNAs and regulatory RNA features were identified by searching the genome for corresponding Ram profiles using INFERNAL v1.1.1 [34]. Signal peptides were identified by Signalp v4.1 [35] and clustered regularly interspaced short palindromic repeats (CRISPR) were detected by using MinCED v0.2.0 included in the Prokka annotation pipeline [36]. Analyses to identify genes that were assigned to transmembrane domains were performed by using tmhmm [37]. Circular maps were created using CGView [38].

## Genome properties

**Fig. 3:**
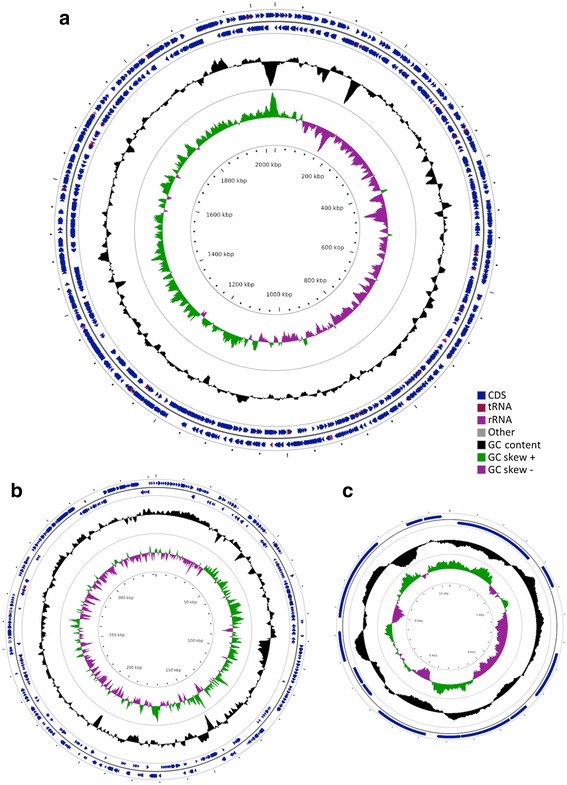
Graphical circular maps of *T. brockianus* GE-1 replicons. The complete genome of *T. brockianus* GE-1 is composed of a single circular chromosome that consists of 2,035,182 bp (**a**) and two circular plasmids, pTB1 (**b**) and pTB2 (**c**). The size of megaplasmid pTB1 is 342,792 bp and 10,299 bp for pTB2. These maps were generated by using CGView [38]. Data shown on those maps will be explained from the inside to the outside: Second circle represents the GC skew of both strands (green for plus strand, purple for minus strand) and the fourth circle shows the GC content. The sixth and seventh circle exhibits the protein-encoding genes for the plus and minus strand as well as RNA features. All tRNAs are highlighted in orange, rRNAs are shown in light purple and other RNAs are represented by a grey color

The complete genome of *T. brockianus* GE-1 is composed of a single circular chromosome that consists of 2,035,182 bp and two circular plasmids, pTB1 and pTB2 (Fig. 3). The megaplasmid pTB1 has got 342,792 bp and the size of pTB2 is 10,299 bp (Table 3). The GC content of each replicon is 67.07 for the chromosome, 65.82 for megaplasmid pTB1 and 70.09 for plasmid pTB2. The *T. brockianus* GE-1 genome comprises 2,511 genes, including 2,458 protein-encoding genes (97.9%), 53 RNAs (2.1%) and 66 pseudo genes (2.6%) (Table 4). In more detail there are 2,053 protein-encoding sequences for the chromosome, 299 for megaplasmid pTB1 and 13 for plasmid pTB2. Classification of genes into the COG functional categories is shown in Table 5.

**Table 3:** Summary of the genome of *Thermus brockianus* GE-1: 1 chromosome and 2 plasmids

Label	Size (Mb)	Topology	INSDC identifier	RefSeq ID
Chromosome	2.035	circular	CP016312	-
pTB1	0.343	circular	CP016313	-
pTB2	0.010	circular	CP016314	-

**Table 4:** Genome statistics

Attribute	Value	% of Total^a^
Genome size (bp)	2,388,273	100.0
DNA coding (bp)	2,217,408	92.9
DNA G + C (bp)	1,597,811	67.0
DNA scaffolds	3	100.0
Total genes	2,511	100.0
Protein coding genes	2,458	97.9
RNA genes	53	2.1
Pseudo genes^b^	66	2.6
Genes in internal clusters	-	-
Genes with function prediction	1,834	73.0
Genes assigned to COGs	1,948	77.6
Genes with Pfam domains	1,736	69.1
Genes with signal peptides	112	4.5
Genes with transmembrane helices	561	22.3
CRISPR repeats	8	0.3

^a^The total is based on either the size of the genome in base pairs or the total genes in the annotated genome

^b^Pseudo genes may also be counted as protein coding or RNA genes, so is not additive under total gene count

**Table 5:** Number of genes associated with general COG functional categories

Code	Value	%age	Description
J	143	5.81	Translation, ribosomal structure and biogenesis
A	0	0.00	RNA processing and modification
K	87	3.54	Transcription
L	106	4,31	Replication, recombination and repair
B	2	0.08	Chromatin structure and dynamics
D	28	1.14	Cell cycle control, Cell division, chromosome partitioning
V	27	1.10	Defense mechanisms
T	71	2.89	Signal transduction mechanisms
M	84	3.42	Cell wall/membrane biogenesis
N	14	0.57	Cell motility
U	18	0.73	Intracellular trafficking and secretion
O	84	3.42	Posttranslational modification, protein turnover, chaperones
C	155	6.31	Energy production and conversion
G	123	5.00	Carbohydrate transport and metabolism
E	207	8.42	Amino acid transport and metabolism
F	70	2.85	Nucleotide transport and metabolism
H	107	4.35	Coenzyme transport and metabolism
I	78	3.17	Lipid transport and metabolism
P	100	4.07	Inorganic ion transport and metabolism
Q	23	0.94	Secondary metabolites biosynthesis, transport and catabolism
R	253	10.29	General function prediction only
S	168	6.83	Function unknown
-	510	20.75	Not in COGs

The total is based on the total number of protein coding genes in the genome

## Insights from the genome sequence

### Comparison of genomes from *T. brockianus* GE-1 and other *Thermus* species

Based on the availability of their finished genomes within the NCBI genome database we compare the *T. brockianus* GE-1 genome with six other *Thermus* species and seven genomes, including *T. thermophilus* HB8, *T. thermophilus* HB27 [39], *T. scotoductus* SA-01 [40], *Thermus* sp. CCB_US3_UF1 [41], *T. oshimai* JL-2 [42], *T. aquaticus* Y51MC23 [43] and *T. parvatiensis* [44]. With 2.38 Mb the genome of *T. brockianus* GE-1 is the largest one of these finished genomes, close to the genomes of *T. oshimai* JL-2 (2.33 Mb), *T. aquaticus* Y51MC23 (2.34 Mb) and *T. scotoductus* SA-01 (2.36 Mb) and much bigger than *Thermus* sp. CCB_US3_UF1 (2.26 Mb), *T. thermophilus* HB8 (2.12 Mb), *T. thermophilus* HB27 (2.13 Mb) and *T. parvatiensis* (2.03 Mb). All of those finished genomes include a chromosome and at least one plasmid. The genome of *T. brockianus* GE-1 consists of one chromosome (2.04 Mb) and two plasmids, including megaplasmid pTB1 (0.34 Mb) and plasmid pTB2 (10 kb). In number and size of those replicons the genome of *T. brockianus* GE-1 is similar to *T. thermophilus* HB8 having a chromosome (1.85 Mb), the megaplasmid pTT27 (0.26 Mb) and the plasmid pTT8 (9.3 kb) as well as *T. oshimai* JL-2 with one chromosome (2.07 Mb), one megaplasmid pTHEOS01 (0.27 Mb) and one plasmid pTHEOS02 (6 kb). Megaplasmids are a common feature of *Thermus* spp., since they were also identified in *T. thermophilus* HB27 (pTT27; 0.23 Mb) and *T. parvatiensis* (pTP143; 0.14 Mb). Due to their thermophilic lifestyle, all finished *Thermus* genomes exhibit a high GC content varying between 64.9% for *T. scotoductus* SA-01 and 69.5% for *T. thermophilus* HB8 with an average value of 67.0% GC content for the genome of *T. brockianus* GE-1.

With its total gene number of 2,511 and 2,458 protein-encoding genes, the genome of *T. brockianus* GE-1 showed high-ranking numbers in comparison to the available genomes, comparable to *T. oshimai* JL-2 (2,580 in total and 2,436 protein-encoding genes), *T. scotoductus* SA-1 (2,511 and 2,458), *T. aquaticus* Y51MC23 (2,484 and 2,325) and higher than *Thermus* sp. CCB_US3_UF1 (2,333 and 2,279), *T. thermophilus* HB8 (2,226 and 2,173), *T. thermophilus* HB27 (2,263 and 2,210) and *T. parvatiensis* (1,573 and 2,190). The genome of *T. brockianus* GE-1 encodes 47 tRNA genes and 6 rRNA genes, similar to most of the other finished genomes. Additionally, eight clustered regularly interspaced short palindromic repeats (CRISPRs) were identified in the genome of *T. brockianus* GE-1, suggesting the presence of a defense mechanism against phage DNA invasion, equivalent to other finished *Thermus* genomes with reported CRISPR sequences, including *Thermus* sp. CCB_US3_UF1 (8), *T. thermophilus* HB8 (11) and HB27 (10) and *T. scotoductus* SA-01 (3) [41].

By whole genome comparison analyses we identified the highest number of protein orthologs in the genomes of *T oshimai* (85.86%) and *T. aquaticus* (85.34%). These two genomes shared 2,156 and 2,143 of the 2,511 total proteins with *T. brockianus* GE-1. Further comparisons revealed 83.07% protein orthologs in comparison to *Thermus* sp. CCB_US3_UF1 (2,086 of 2,511), 82.44% to *T. scotoductus* (2,070 of 2,511), 81.96% to *T. thermophilus* HB8 (2,058 of 2,511) and 81.76% to *T. thermophilus* HB27 (2,053 of 2,511). With 1,661 and 66.15% we identified the lowest numbers of protein orthologs between *T. parvatiensis* and *T. brockianus* GE-1.

The number of total and protein-encoding genes on megaplasmid pTB1 were 314 and 299 as well as 13 for both in case of pTB2. Especially, the number of genes on the megaplasmid pTB1 is much higher compared to other available megaplasmids, since their total gene numbers vary between 150 (*T. parvatiensis*) and 251 (*T. thermophilus* HB8). These differences are explicable by the smaller size of both megaplasmids (0.14 Mb for pTP143 and 0.26 MB for pTT27) in comparison to pTB1 (0.34 Mb). In contrast, the size of pTB2 (10 kb) is smaller than most other additional plasmids, which were reported to be 6–60 kb.

General metabolic pathways were investigated by KEGG analysis and revealed complete sets of genes for glycolysis, gluconeogenesis, citrate cycle, pentose phosphate pathway as well as genes involved in the lipid-, nucleotide-, amino acid-, cofactor- and vitamin-metabolism. For nutrient uptake, we identified 14 ABC transporters. All of these genes were localized on the chromosome of *T. brockianus* GE-1.

### Xylan degradation pathway

Thermophilic bacteria like *T. brockianus* are of great industrial relevance, because they produce heat-stable and heat-active enzymes, so called thermozymes that perfectly match harsh process conditions. With regard to biocatalysts with a great potential for biotechnological processes, e.g. biorefinery, we identified sequences encoding putative lipases, subtilisin-like proteases, glucosidases and galactosidases in the genome of *T. brockianus* GE-1. The observed ability of this strain to degrade xylan-rich substrates and the identification of the responsible xylanase Xyn10 in our previous study directed our interest towards the investigation of the xylan degradation pathway by performing an analysis of the whole genome sequence of *T. brockianus* GE-1 [14]. Interestingly, there is no homologue xylanase sequence detectable in any other *Thermus* genome that is currently available. Thus, the amino acid sequence of the xylanase Xyn10 from *T. brockianus* strain GE-1 displays the highest identity (57%) to a 1,4-β xylanase from *Streptomyces* sp. NRRL WC-3723. These two organisms are not closely related but it can be hypothesized that due to similar environmental conditions an ancestor of *T. brockianus* obtained the coding sequence of *xyn10* by horizontal gene transfer. Another interesting fact to consider is that the localization of the corresponding gene *xyn10* was not detected on the bacterial chromosome like other hydrolases, including lipases, peptidases and ATPases.

**Fig. 4:**
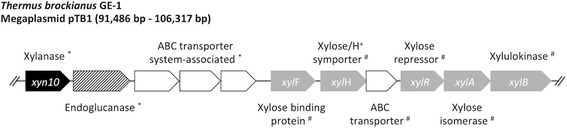
Genomic organization of genes encoding proteins for xylan and cellulose degradation as well as xylose metabolism located on the megaplasmid pTB1 of *T. brockianus* GE-1. Sizes, localization and orientation of the genes on megaplasmid pTB1 section are displayed proportionally. All genes highlighted with a star are not detectable in any other *Thermus* spp. genome except *T. brockianus* GE-1. Genes marked with a diamond are conserved in *Thermus* spp. ABC transporter system associated genes include sugar ABC transporter substrate-binding protein and two sugar ABC transporter permeases

Further investigations of the genomic region revealed an unique set of genes related to cellulose degradation and xylose metabolism adjacent to *xyn10*, including a putative endoglucanase as well as membrane ABC sugar transporter encoded by *xylH*, *xylF* encoding the xylose binding protein, the xylose repressor encoding gene *xylR*, the xylose isomerase encoding gene *xylA* and the gene *xylB* coding for a xylulokinase (Fig. 4)*.* Especially the latter ones are of great importance, since XylA catalyzes the first step of the xylose metabolism by isomerization of xylose to xylulose, while XylB is responsible for the second step, phosphorylating xylulose to xylulose-5-phosphate [45, 46]. These xylose metabolism-related genes are conserved in other *Thermus* spp. genomes and a similar set has been described for plasmid pVV8 in the genome of *T. thermophilus* HB8 [46, 47]. Genome comparison studies of pVV8 and pTB1 revealed 75% identity of the genomic region encoding the xylose metabolism-related genes. However, the xylanase, the endoglucanase and the ABC transporter system-associated genes are described for the first time in a *Thermus* species genome. These genes showed highest similarities (43% to 57%) to distantly related genera including *Streptomyces* and *Alicyclobacillus*. The absence of a β-xylosidase in the genome of *T. brockianus* GE-1 is explicable by the β-xylosidase side activity of xylanase Xyn10, thus breaking down xylan directly to D-xylose [14]. Altogether the localization of this whole set of genes on the megaplasmid pTB1 in combination with the non-essential character of the xylan degradation pathway for the lifestyle of *T. brockianus* GE-1 indicates that *T. brockianus* GE-1 has gained the xylanolytic ability as a beneficial advantage, probably via horizontal gene transfer in an ancestor of *T. brockianus*. In accordance to the bioconversion of lignocellulosic biomass, another interesting point to consider is the identification of a putative endoglucanase. By linking genes encoding endoglucanase and xylanase on its megaplasmid pTB1, these enzymes might act in concert in a synergistically fashion. Especially, in regard to a recent report of an evolved strain of *T. thermophilus* co-utilizing xylose and glucose [16], *T. brockianus* GE-1 and its unique genomic linkage of sequences encoding key enzymes for xylan and cellulose degradation as well as for xylose metabolism seems to be of great interest for biotechnical applications and thus will be examined in prospective studies.

## Conclusions

*Thermus* spp. and their extremozymes are of great interest for a wide set of industrial applications. Here we present the first whole genome sequence of *T. brockianus* GE-1, providing further insights into the biotechnological potential of the genus *Thermus* spp. in general and *T. brockianus* GE-1 specifically. The genome of *T. brockianus* GE-1 consists of a chromosome and two plasmids, including the megaplasmid pTB1. Sequences coding for essential metabolism pathways like glycolysis, gluconeogenesis, pentose phosphate pathway or citrate cycle were assigned to the bacterial chromosome just as well as sequences encoding industrial relevant enzymes, including galactosidases, glucosidases, lipases and subtilisin-like proteases. These novel extremozymes will be targets of prospective characterization studies to prove their industrial relevance. However, localization of gene *xyn10* coding for a previously described xylanase from *T. brockianus* GE-1 was not detected on the chromosome but on the megaplasmid pTB1 adjacent to sequences encoding key enzymes for cellulose degradation and xylose metabolism. Thus, in accordance to a reported β-xylosidase side activity of xylanase Xyn10 the complete breakdown of xylan to D-xylose is genetically linked to the xylose metabolism in the genome of *T. brockianus* GE-1. These findings are consistent with the described xylanolytic activity of *T. brockianus* GE-1. The described combination of the identification of novel sequences encoding putative biocatalysts on the one hand and the description of a xylanolytic degradation pathway on the other hand emphasize the importance of *Thermus* spp. as promising sources of extremozymes with potential industrial value.

## Abbreviations

COG: Clusters of Orthologous Groups
CRISPR: Clustered regularly interspaced short palindromic repeats
QV: Quality value
